# Antimicrobial efficacy of composite irrigation solution against dominant pathogens in seawater immersion wound and *in vivo* wound healing assessment

**DOI:** 10.3389/fmicb.2023.1188373

**Published:** 2023-05-25

**Authors:** Xin Wang, Jie Tan, Shenpeng Ni, Dengyun Zhou, Baolin Liu, Qiang Fu

**Affiliations:** ^1^School of Health Science and Engineering, University of Shanghai for Science and Technology, Shanghai, China; ^2^Shanghai Technical Service Platform for Cryopreservation of Biological Resources, Shanghai, China; ^3^Shanghai Co-Innovation Center for Energy Therapy of Tumors, Shanghai, China; ^4^Department of Orthopedics, Shanghai General Hospital, Shanghai Jiaotong University, Shanghai, China

**Keywords:** irrigation solution, seawater immersion, dominant pathogen, antimicrobial efficacy, wound healing

## Abstract

Seawater immersion wound is inevitably accompanied by bacterial infection. Effective irrigation is critical for bacterial infection prevention and wound healing. In this study, the antimicrobial efficacy of a designed composite irrigation solution against several dominant pathogens in seawater immersion wounds was evaluated, and *in vivo* wound healing assessment was conducted in a rat model. According to the time–kill result, the composite irrigation solution exhibits excellent and rapid bactericidal effect against *Vibrio alginolyticus* and *Vibrio parahaemolyticus* within 30 s of treatment while eliminating *Candida albicans*, *Pseudomonas aeruginosa*, *Escherichia coli,* and the mixed microbes after 1 h, 2 h, 6 h, and 12 h of treatment, respectively. Significant bacterial count reduction of *Staphylococcus aureus* was observed after 5 h treatment. In addition to its skin non-irritating attribute, the *in vivo* wound healing results further demonstrated that the irrigation solution showed high repair efficiency in the skin defect model inoculated with the mixed microbes. The wound healing rate was significantly higher than that of the control and normal saline groups. It could also effectively reduce the number of viable bacteria on the wound surface. The histological staining indicated that the irrigation solution could reduce inflammatory cells and promote collagen fibers and angiogenesis, thereby promoting wound healing. We believed that the designed composite irrigation solution has great potential for application in the treatment of seawater immersion wounds.

## Graphical abstract

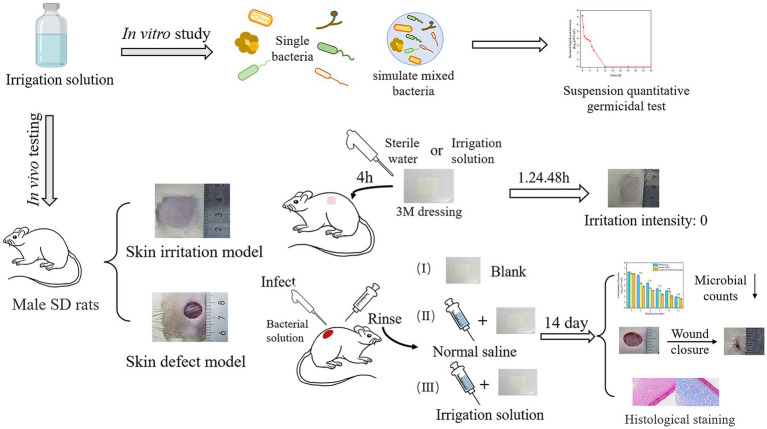

## Highlights

– Several dominant pathogens in seawater immersion wound were taken as the study object.
– The irrigation solution has good antimicrobial efficacy on selected pathogens.
– The irrigation solution can promote wound healing and tissue regeneration.
– The irrigation solution has no irritation to the skin.

## 1. Introduction

Nowadays, the exploration of the ocean and marine resources became more and more frequent, and casualties falling into the sea during the naval war, marine operations, dangerous marine life attacks, or other accidents would inevitably occur. Seawater immersion is the most important difficulty that wounded personnel often encounter. Due to its characteristics of high salt content, low temperature, high osmolality, and large microorganism population, tissue edema and necrotic infection may occur rapidly after the skin trauma is soaked by seawater and develop into seawater immersion injury (Yan et al., 2016; Fang et al., 2020). In addition to the dominant pathogens commonly found in terrestrial wounds, such as *Staphylococcus aureus*, *Escherichia coli*, *and Pseudomonas aeruginosa*, a large number of pathogenic Vibrio species have been observed in marine injuries, specifically *Vibrio alginolyticus*, *Vibrio parahaemolyticus*, and *Vibrio vulnificus,* which can inhibit wound repair and lead to serious and even life-threatening infections (Diaz, 2014). As a result, the infection rate of such wounds is obviously higher than that of terrestrial wounds, and the mortality is 5–10 times higher than that of terrestrial wounds (Liu et al., 2020). Therefore, controlling the microbial bioburden is a key factor in preventive and therapeutic wound management.

According to the “TIME” principle (tissue, infection/inflammation, moisture and edge) of modern wound care, cleansing and keeping moist of the wound are very important for wound healing (Davis et al., 2017). Many wound dressings have been created to maintain a moist environment at the wound interface, acting as a barrier to microorganisms and removing excess exudates. For example, due to their excellent biocompatibility, stimuli responsibility, controllable mechanical strength, and porous structure, hydrogel-based composites have been a kind of promising wound dressing (Wang et al., 2021; Li et al., 2022b). It should be noted that among the common treatment procedure for contaminated open injury, irrigation and debridement are essential and need to be properly conducted before the coverage with a suitable wound dressing. Timely wound irrigation can effectively remove cell debris and infecting microorganisms, lessening bacterial replication on the site, and reducing the chance of infection (Davis et al., 2017). The ideal irrigation solution should not only have a good sterilization effect but also have the characteristics of low concentration, good stability, low irritation, low cost, safety, and so on (Chen et al., 2021). Various irrigation solutions are currently being used, for example, sterile saline solution was the most frequently used fluid (Trevillion, 2008), followed by povidone-iodine (Martin et al., 2022), chlorhexidine (Willigenburg et al., 2023), hydrogen peroxide (Mohammadi et al., 2013), and antibiotic solutions (Siafaka et al., 2016; Ameeduzzafar et al., 2018). However, considering that the antibacterial properties of these irrigation solutions were mostly evaluated using *Escherichia coli* and *Staphylococcus aureus* (Zhou et al., 2017; Aubert-Viard et al., 2019), which was different from the microbiota composition of the seawater immersion wound, common medical irrigations have little effect when the affected area is immersed in seawater, and rarely, irrigations have been reported for the treatment of seawater-immersed wounds. Hence, designing a suitable irrigation solution for the clinical infection control of seawater immersion wounds is critical, and it would be beneficial for the healing period.

Considering that the number of bacteria in the wound after irrigating is proportional to the rate of wound infection, the evaluation of antimicrobial efficacy is very important in the development of an irrigation solution. Generally, the diameter of the inhibition zones and the time–kill curve can be used to evaluate the antibacterial activity *in vitro* (Hu et al., 2021). For example, the bactericidal property of trimethyl chitosan chloride-based postoperative irrigation solution against bacteria *S. aureus* and *E. coli* was evaluated using the inhibition area values (Abueva et al., 2022). A quantitative suspension method was used to evaluate the antimicrobial efficacy of NaClO/HClO wound irrigation solutions against bacteria presented in low-level wound infections, that is, *S. aureus* and *Pseudomonas aeruginosa* (Severing et al., 2019). In the evaluation of common antimicrobial solutions used for breast implant soaking and breast pocket irrigation, planktonic gram-positive and gram-negative bacterial strains were exposed to triple antibiotic solution (TAB) and/or 10% povidone-iodine (PI) for up to 10 min in a bacterial time–kill assay (Jewell et al., 2021). Meanwhile, the antimicrobial efficacy of irrigation solution also can be evaluated *in vivo*, for example, a porcine wound model was employed to evaluate the effects of PHMB wound irrigation solution on chronic wounds, and methicillin-resistant *S. aureus* (MRSA) biofilms were used (Davis et al., 2017). The efficacy of ethylenediaminetetraacetic acid (EDTA) irrigation solution against drug-resistant bacteria was tested in a rat wound infection model (Deng et al., 2019). In this case, wound healing promotion can also be evaluated by wound closure, the number of bacteria in wounds, as well as histology and immunohistochemistry, providing more profound information for future clinical translation (Abueva et al., 2022). Apart from animal experiments, which is the gold standard for testing the safety and efficacy of therapeutics for centuries, the possibility of providing controlled and repeatable *in vitro* skin models using organoids, 3D bioprinting, and skin-on-a-chip platforms has also been discussed, which presented a new opportunity to evaluate the effect of inflammation stress on skin tissue at a cellular level (Sutterby et al., 2022).

Herein, to determine the application potential of a designed composite irrigation solution for seawater immersion wound management, the antimicrobial efficacy against dominant pathogens in seawater immersion wound, that is, four common pathogenic microbes of terrestrial wounds (*S. aureus, E. coli, P. aeruginosa,* and *Candida albicans*), two marine vibrios (*V. alginolyticus* and *V. parahaemolyticus*), and simulated marine mixed microbes, was conducted *in vitro*, and the *in vivo* wound healing assessment was evaluated using a rat wound infection model (see Table 1).

**Table 1:** Various strains for experiments.

Species	Acronym	Strain number	Source
*Staphylococcus aureus*	*S. aureus*	ATCC-6538	SHU
*Escherichia coli*	*E. coli*	ATCC-25922	SHU
*Vibrio parahaemolyticus*	*V. parahaemolyticus*	ATCC-17802	NICPBP
*Vibrio alginolyticus*	*V. alginolyticus*	KY684259	ECSFRI CAFS
*Pseudomonas aeruginosa*	*P. aeruginosa*	ATCC-9027	FMSETRDC
*Candida albicans*	*C. albicans*	ATCC-10231	FMSETRDC

SHU, Shanghai University; NICPBP, China National Institute for the Control of Pharmaceutical and Biological Products; ECSFRI CAFS, East China Sea Fishery Research Institute, Chinese Academy of Fishery Sciences; FMSETRDC, Guangdong Province Food Microbial Safety Engineering Technology Research and Development Center.

## 2. Materials and methods

### 2.1. Reagents and materials

Levofloxacin (LVX, 98%) was purchased from Shanghai Macklin Biochemical Co., Ltd. (Shanghai, China). Peroxide (H_2_O_2_, 3%) was purchased from Guangdong Hengjian Pharmaceutical Co., Ltd. Polyhexamethylene biguanide (PHMB, 99%), sodium chloride (AR), soybean lecithin (BR), glacial acetic acid, paraffin, formaldehyde, xylene, absolute ethanol, and ammonia water were obtained from Sinopharm Chemical Reagent Co., Ltd. Chitosan (CS, DD: 90%, Mw:700,000–800,000) was obtained from Shanghai Lanji Biotechnology Co., Ltd. Sodium thiosulfate pentahydrate, Tween 80, magnesium sulfate (AR), and bovine serum albumin (BR) were purchased from Sangon Biotech Co., Ltd. (Shanghai, China). Tryptone physiological solution (TPS) (BR) was bought from Guangdong Huan Kai Microbial Technology Co., Ltd. Hematoxylin, eosin, BASO, and neutral resin were purchased from Beijing Solarbio Technology Co., Ltd. Masson trichromatic staining solution was bought from Beijing Huayueyang Biotechnology Co., Ltd. Then, 3% sodium chloride alkaline peptone broth, 3% sodium chloride soybean–casein digest agar, thiosulfate–citrate–bile salts–sucrose agar, nutrient broth, nutrient agar, Sabouraud dextrose broth, potato dextrose agar (PDA), cation-adjusted Mueller–Hinton broth (CAMHB), and Mueller–Hinton Agar were acquired from Qingdao Hope Biotechnology Co., Ltd.

### 2.2. Preparation of composite irrigation solution

For the preparation of composite irrigation solution, CS and LVX have dissolved in 1% and 2% glacial acetic acid solutions, respectively. PHMB was dissolved in deionized water and 3% H_2_O_2_ was diluted with sterile water. Subsequently, they were mixed in different volumes so that the final concentration of each component was 10 μg/mL LVX, 80 μg/mL H_2_O_2_, 10 μg/mL PHMB, and 320 μg/mL CS. After that, 1 mol/L L-tartaric acid and NaHCO_3_ were used as acid–base regulators, and the pH of the irrigation solution was adjusted to 4. After preparation, the irrigation solution was placed in a refrigerator at 4°C for sealed storage.

### 2.3. Antimicrobial efficacy: *in vitro* study

#### 2.3.1. Preparation of microbial suspension

The microbial suspension of each strain was prepared using the direct bacterial suspension method (Li et al., 2021). Activated *V. alginolyticus* and *V. parahaemolyticus* were inoculated onto TCBS medium plates, *S. aureus, E. coli,* and *P. aeruginosa* onto nutrient agar plates, and *C. albicans* onto PDA plates, respectively. After incubating for 18–24 h, single colonies of *V. alginolyticus* or *V. parahaemolyticus* were inoculated into 3% NaCl-CAHMB liquid medium, and *S. aureus, E. coli, P. aeruginosa,* or *C. albicans* were inoculated into CAHMB liquid medium. After incubating at 37°C for 18–24 h in a 180 rpm shaker, the concentration of the microbial suspension was adjusted to about 5 × 10^9^ CFU/mL. Meanwhile, based on literature research and discussion with clinicians about the pathogens related to seawater immersion wound, the microbial suspension was prepared according to the following proportions: 28% of *V. alginolyticus*, 28% of *V. parahaemolyticus*, 2% of *S. aureus*, 20% of *E. coli*, 20% of *P. aeruginosa,* and 2% of *C. albicans,* so as to simulate the dominant pathogens in seawater immersion wound (Hörmansdorfer et al., 2000).

#### 2.3.2. Suspension quantitative germicidal test

The suspension quantitative germicidal test was conducted to investigate the time-dependent antibacterial activity of the composite irrigation solution (Meade and Garvey, 2018). First, 5 mL of the microbial suspension and 5 mL of 3% bovine serum albumin were added to a 150 mL sterile conical flask, followed by 40 mL of the composite irrigation solution, and incubated in a 110 rpm shaker at 37°C. Then, 0.5 mL was taken at 30 s, 1, 2, 3, 4, 5, 6, 12, 24, and 36 h in a 10 mL sterilized test tube, and 4.5 mL of neutralizer (80 mg/mL sodium thiosulfate, 5 mg/mL Tween 80, 1 mg/mL lecithin, 12 mg/mL magnesium sulfate, and 0.1% TPS) was added. After acting for 10 min, 100 μL neutralized product was taken for further colony counting on their optimal plates, and 3% sodium chloride soybean–casein digest agar was selected as the counting medium for mixed bacteria based on a preliminary experiment.

### 2.4. Wound healing promotion: *in vivo* testing

#### 2.4.1. Experimental animals and ethics approval

A total of 12 SD male rats (250 g) were used in animal experiments, including skin irritation and wound healing tests. All the rats were allowed to acclimate (at least 1 week) to the laboratory conditions before testing and all operations were performed under sterile conditions.

The study and all procedures were approved by the Institutional Animal Care and Use Committee Guidelines of Jiangsu University (UJS-IACUC-2021041601). All study methods were in accordance with China’s regulations on experimental animal usage, which were consistent with Animal Research: Reporting of *In Vivo* Experiments (ARRIVE) guidelines.

#### 2.4.2. Skin irritation testing

One complete skin irritation test was conducted with slight modifications in reference to the method of Mekonnen et al. (2019). Three male SD rats were selected, and the hair on both sides of the spinal column was removed 24 h before the experiment. On the next day, 0.5 mL of composite irrigation solution was dropped onto a 2.5 cm × 2.5 cm 3 M dressing on one side of the depilated skin for 4 h and sterile water as a blank control on the other side. The skin reactions were observed at 1, 24, and 48 h after removal of the test substances. The formation of skin erythema and edema was scored, and the grade of skin irritation intensity of the test object on animals was evaluated (Li et al., 2022a).

#### 2.4.3. Rat infection and irrigation

The skin defect model was used to simulate the clinical situation of soft-tissue wound. Male SD rats (approximately 250 g) were randomly divided into three groups, with three rats in each group. Before surgical procedures, the mice were gas-anesthetized, the area around the surgical field was shaved off and disinfected with ethanol, and a circular full-thickness wounds with a diameter of 10 mm were prepared on both sides of the back, respectively, in reference to the methods of Lin et al. (2019). The wound was then inoculated with 100 μL of 1 × 10^8^ CFU/mL mixed bacterial suspension.

After 24 h microbial attack on the wound, the following treatments were conducted by the same researcher (NSP): (I) the wound surface was directly covered with 3 M dressing. (II) The wound was rinsed with 60 mL of 0.9% normal saline by a syringe with maximal manual pressure and then covered with 3 M dressing (Fernandez et al., 2007). (III) The wound was rinsed with 60 mL of composite irrigation solution and then covered with 3 M dressings. All the dressings were changed every 2 days and the wounds were photographed.

#### 2.4.4. Wound closure measurement

The wounds in the different groups were photographed at different time points (0, 1, 3, 5, 7, 9, 11, and 14 days) using the digital camera and measured by ImageJ v1.53e (National Institute of Mental Health, United States) software. The wound area was calculated from the photograph and the scale, and the healing rate was calculated according to the following equation:


$$H=\left(S_{0}-S_{X}\right)/S_{0}*100\%$$

where *S_X_* is the rat wound area on day *X*, and *S_0_* was the original wound area on day 0, respectively.

#### 2.4.5. Microbial count

This experiment was evaluated with slight modifications based on the method of Yu et al. (2020). The wound exudates were collected using sterile cotton swabs infiltrated with sterile normal saline. Then, it was dispersed in 1 mL of sterile saline solution and diluted in gradient, and 100 μL was coated on the 3% sodium chloride soybean–casein digest agar plate, cultured at 37°C for 48 h, and then, the colony was counted.

#### 2.4.6. Histological evaluation

Histological evaluation was conducted based on the method of Shu et al. (2022). The mice were sacrificed on days 7 and 14, and the tissues at the wound site were cut, fixed in 10% formalin for 48 h, and dehydrated with different concentrations of anhydrous ethanol; then, the samples were clarified to a clear state and embedded in paraffin. The slices were cut with a thickness of 4–7 μm. All sections were stained with hematoxylin and eosin (H&E) and Masson trichrome stain for histological analysis.

### 2.5. Statistical analysis

The colony counts for the suspension quantitative sterilization test and the total number of microbes on the wound surface were set up in three parallel experiments. Excel 2016 (Microsoft, Redmond, United States) was used for data processing; SPSS 18.0 (SPSS, Chicago, United States) was used for one-way ANOVA and identifying significant differences, and *p*-values of ^*^(<0.05), ^**^(<0.01), and ***(<0.001) were considered statistically significant. Origin 2018 software (OriginLab, Northampton, United Kingdom) was used for drawing graphics.

## 3. Results and discussion

### 3.1. Antimicrobial efficacy of the composite irrigation solution on different pathogenic microorganisms

**Figure 1:**
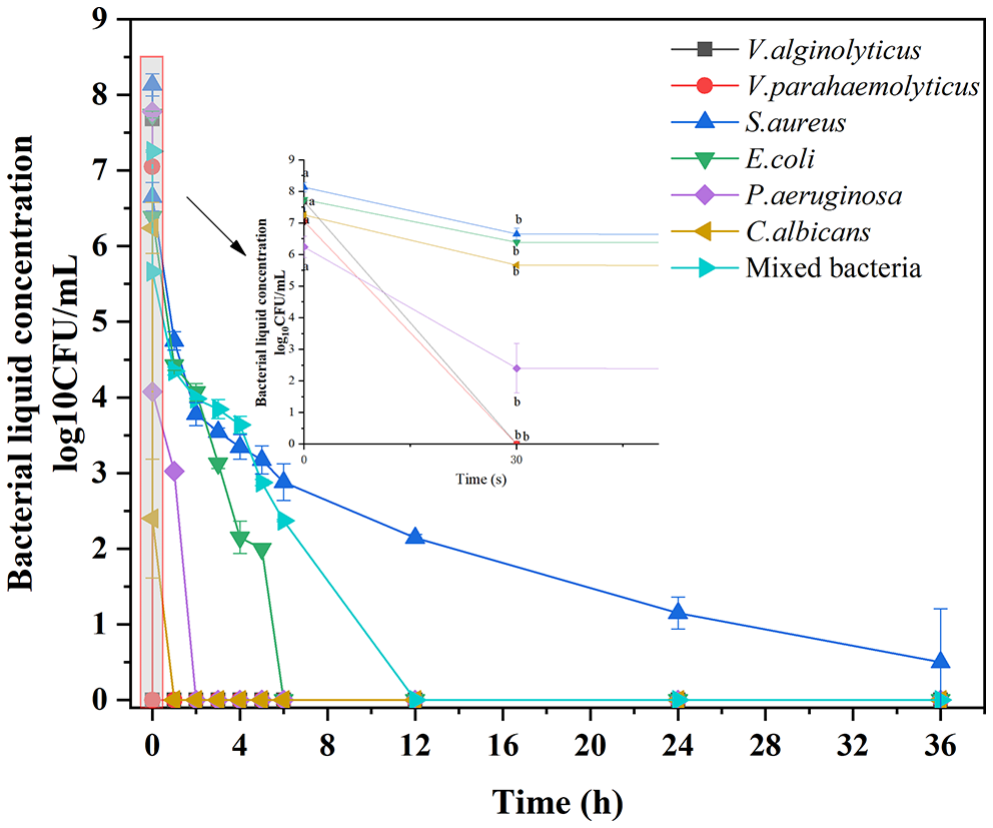
Antimicrobial effect of the composite irrigation solution on different pathogenic microbes: time–kill curve.

To characterize the antimicrobial activity of the composite irrigation solution against the selected pathogenic microbes, time–kill assays were performed, and the killing effects at various time intervals are shown in Figure 1. The irrigation solution appeared to exhibit excellent and rapid bactericidal effect against *V. alginolyticus* and *V. parahaemolyticus,* and no growth was observed after 30 s of exposure. While after 1 h and 2 h of treatment, it was also effective at eliminating *C. albicans* and *P. aeruginosa,* respectively. When the exposure time was prolonged to 6 h and 12 h, the growth of *E. coli* and the mixed microbes can be completely limited, and over 5 log_10_ reductions of these two strains occurred at 4 and 6 h, respectively, indicating significant microbial reduction effect. Only the *S. aureus* cannot be eradicated during the treatment, but a significant reduction of bacterial counts (>5 log_10_ reduction) was observed after 5 h treatment, showing acceptable antimicrobial effects (GB/T 38502-2020). This significant microbial reduction effect was related to the major bacteriostatic component in the irrigation solution, that is, the positively charged PHMB, which can interact with negatively charged phospholipid groups on the bacterial cell wall and destroy the cell membrane stability, eventually leading to microbial death (Llorens et al., 2015). As a fluoroquinolone antibiotic, levofloxacin can inhibit the bacterial DNA gyrase, thereby also achieving the antibacterial effect (Siddiquee et al., 2021). Additionally, H_2_O_2_ also can reduce cell integrity and kill bacteria by reacting rapidly with the membrane proteins of bacterial cells (Jiang et al., 2022). As a result, a synergistic antibacterial effect was achieved in this composite irrigation solution.

On the other hand, although Abueva et al. (2022) evaluated the bactericidal characteristics of trimethyl chitosan-saline irrigation solution by the diameter of the inhibition circle, the inhibition effects of different irrigation solutions were only judged using *S. aureus* and *E. coli*, which was incompatible with the actual wound flora. Meanwhile, there were few reports of irrigations regarding the treatment of marine bacterial infections (Diaz and Lopez, 2015). Inspired by the study conducted by Lv et al. (2023), who employed not only common bacteria, such as *E. coli*, *S. aureus*, methicillin-resistant *S. aureus*, and *V. parahaemolyticus*, but also marine bacteria (*V. vulnificus* and *P. aeruginosa*) to evaluate the antibacterial effect of hydrogel on seawater-immersed wounds, a mixed microbial suspension was prepared to simulate the dominant pathogenic flora of seawater immersion wound in our study, therefore, ensuring that the irrigation solution was suitable for further practical application. On the other hand, although the phenomenon of colony number reduction and then growth often was observed in the time–killing test of *P. aeruginosa* when evaluating the antibacterial effect of ciprofloxacin, levofloxacin, and ofloxacin (Sihotang et al., 2022), this did not occur in our study, and a complete eradication was reached after 2 h treatment, proving its viability as a wound irrigation solution to reduce the microbial load.

### 3.2. *In vivo* skin irritation assessment

**Figure 2:**
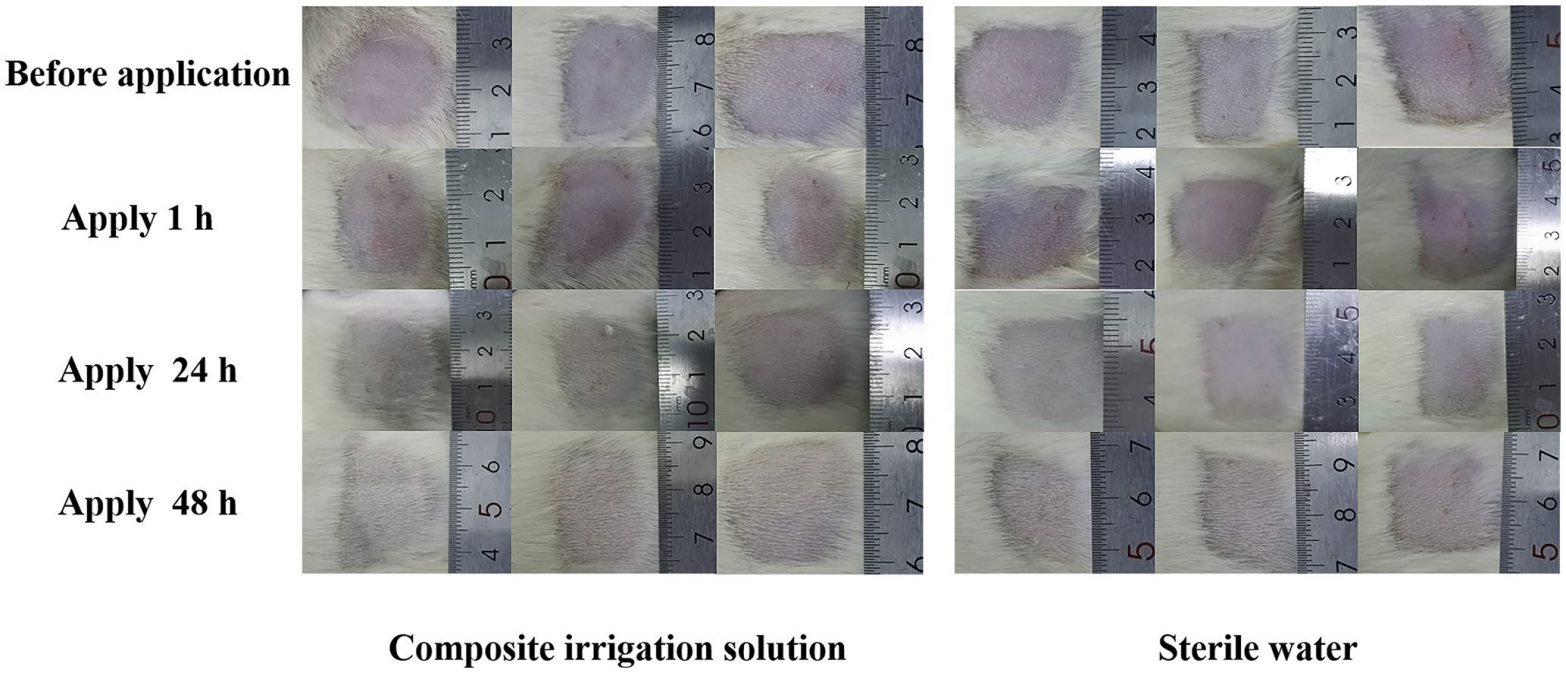
Representative images of SD rats’ skin before and after the application of the composite irrigation solution.

As an acute inflammatory response occurred immediately after stimulation, skin irritation can be rated by the severity of erythema (redness), edema (swelling), itching, and discomfort (Haridas and Rosemary, 2019). After the application of the composite irrigation solution, the skin irritation of the SD rats at 1, 24, and 48 h was evaluated. As shown in Figure 2, no erythema and edema were observed in the skin of the composite irrigation solution group, and the skin condition of SD rats was not significantly different from that of the control group (treated with sterile water). The average skin irritation response score of the composite irrigation solution was 0, and the composite irrigation solution was rated as non-irritating to the skin of animals according to the experimental standard of Li et al. (2022a). This may be explained by that the compounds used in this irrigation solution all showed lower skin irritation effects. For example, as a green oxidant, H_2_O_2_ has no irritation at low concentrations (Zatorska et al., 2021). Mao et al. (2022) evaluated the skin irritation effect of CS and CS-st-HA-Pu film on mice, and no erythema and edema were observed. Bao et al. (2021) also reported that LVX-loaded CS/hyaluronic acid film for ocular administration resulted in no intraocular irritation. As for polyhexanide (PHMB), it showed low host cell cytotoxicity and proved to be well tolerated on the skin (Davis et al., 2017).

### 3.3. Wound closure performance

**Figure 3:**
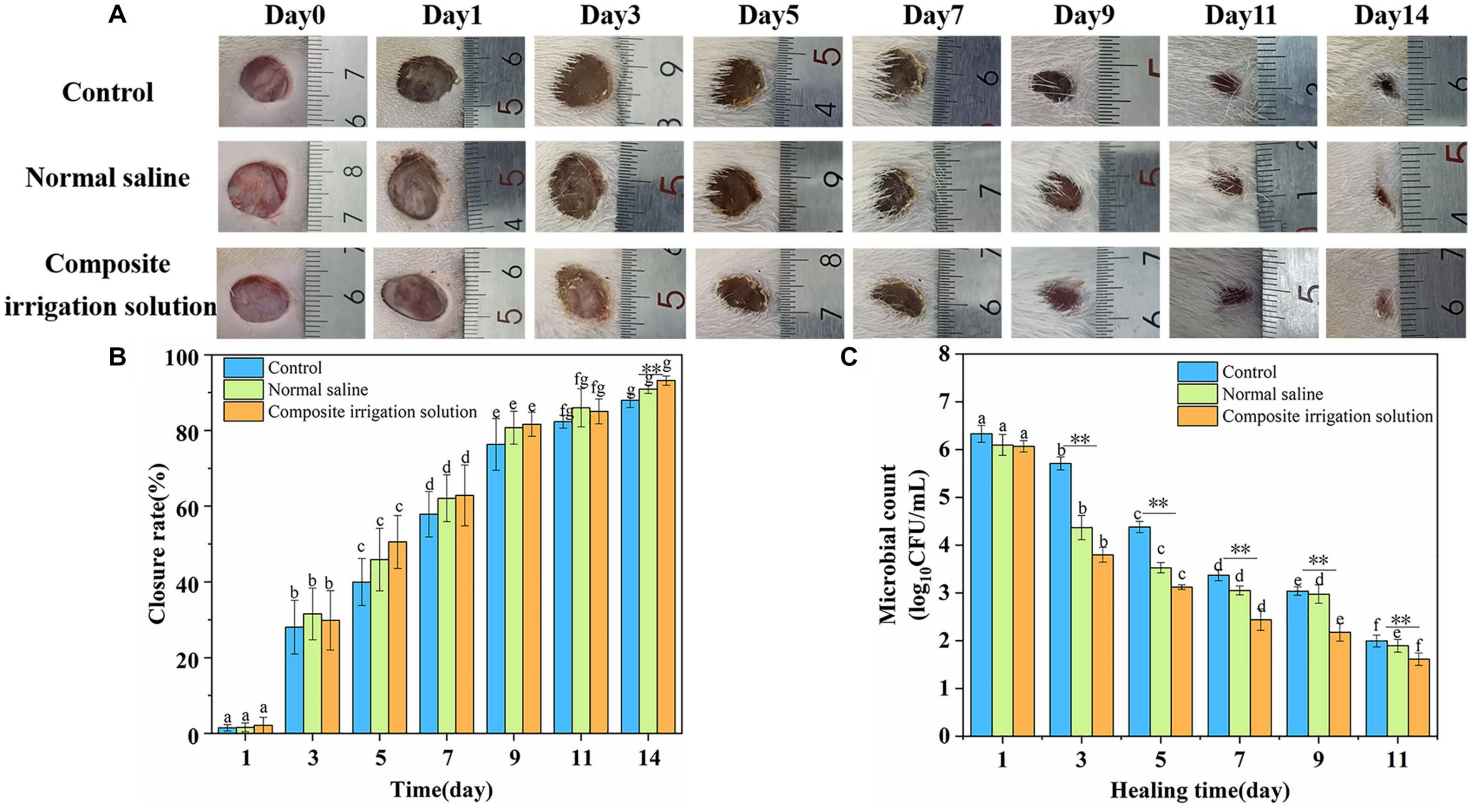
*In vivo* antibacterial activity and wound closure performance of composite irrigation solution. **(A)** Visual images of the healing process upon mixed microbial infected wounds of SD rats with different treatments. **(B)** Change of wound closure rate of SD rats over time. **(C)** Changes of total microbial count on wound surface of SD rats over time. The error bars represent the standard deviation for three measurements (*n* = 3). ^**^Represents intra-group differences (^**^represents very significant *p* < 0.01); a–g represented the differences among groups (*p* < 0.05).

The process of wound healing is complex and involves hemostasis, inflammation, migration, proliferation, and tissue remodeling (Patel et al., 2022). The wound healing images of the mixed microbial infected SD rats’ wounds during the 14 day period are shown in Figure 3A, and the corresponding healing rate is compared in Figure 3B. As shown in Figure 3A, compared with the bright red wound after modeling, the wound’s color and area changed significantly with the prolongation of time. One day after inoculation with the microbial suspension, although the wounds of the three treatments all became darker, the wounds treated with normal saline or composite irrigation solution all showed relatively lighter color, especially the composite irrigation solution group, indicating lower infection formation. On the 3^rd^ day, the wound areas in the two irrigation treatments were obviously reduced, while the scab layer of the control group became thicker and brown, indicating higher inflammation and sluggish wound healing. After the 5^th^ day, the effectiveness of the composite irrigation solution on wound healing was more visible, exhibiting obvious area contraction, followed by the normal saline group. After handling for 14 days, the composite irrigation solution treated wound was almost closed and more hair coverage could be observed, while the other two treatments showed slower wound healing effects.

The wound closure rate was in accordance with the change of wound image. As shown in Figure 3B, the wound closure rates of SD rats in each treatment group increased significantly with the extension of time. The wound closure rate of the irrigation groups was similar to that of the control group, that is, around 29.87 ± 6.04 and 31.55% ± 6.83% in the normal saline group after treating for 3 days. However, the situation changed significantly on the 5^th^ day, and the wound healing rate of the composite irrigation solution group (50.56%) was much higher than that of the control group (39.97%) and the normal saline group (45.88%), respectively, indicating a faster wound healing effect. On the 7^th^ day, the wound closure rates in the two irrigation groups were already over 60% and those in the composite irrigation solution group were still slightly higher than the normal saline group. On the 14^th^ day, the wound closure rate in the composite irrigation solution group was 93.17% and significantly higher than the control and normal saline groups (*p* < 0.01). These results suggested that the composite irrigation solution has better wound healing performance during the early stage of wound healing.

In addition, it was suggested that debridement and irrigation treatment can reduce the positive rate of bacteria in contaminated open wound (Hao and Peng, 2021). Therefore, to further evaluate the *in vivo* antimicrobial ability of the composite irrigation solution, residual bacteria from wound sites were collected and cultured, and the result is shown in Figure 3C. The results indicated that the irrigation treatment was helpful to remove the microbes on the wound surface during the wound healing process. For example, the log_10_CFU/mL of the normal saline and the composite irrigation solution groups was 6.09 and 6.06, respectively, just 1 day after treatment, while that of the control was much higher (6.33). With the prolongation of healing time, the number of viable microbes decreased significantly, and the composite irrigation solution was more effective in reducing the number of pathogens on the wound surface. On the 3^rd^ day, the colony count of the control and normal saline group was 5.71 and 4.36 log_10_CFU/mL, respectively, while that of the composite irrigation solution group obviously decreased to 3.79 log_10_CFU/mL. Meanwhile, although normal saline can flush away the microbes on the wound surface, it was not as effective in the later stages of healing. Nine days later, the colony count of the normal saline group (2.97 log_10_CFU/mL) was similar to that of the control (3.04 log_10_CFU/mL) and was significantly higher than the composite irrigation solution group (2.18 log_10_CFU/mL). These results indicated that the composite irrigation solution had excellent antibacterial ability *in vivo*.

The good wound closure performance of the designed irrigation solution was mainly attributed to the combination of excellent bactericidal effect of antimicrobial compounds such as CS, LVX, H_2_O_2_, and PHMB in it, which can synergistically inhibit the viable microbes in the contaminated wound, thereby promoting wound regeneration. In addition to its good antimicrobial property, chitosan was known as a biocompatible, biodegradable, and non-toxic polymer and has been suggested to be used as an alternative solution to EDTA in final root canal irrigation (Antunes et al., 2020). In another report, the combination of chitosan and chlorhexidine was proved to have better antimicrobial efficacy and could be used as an alternative to NaOCl for endodontic infections (Jaiswal et al., 2017). Meanwhile, PHMB solution also exhibited potent antimicrobial properties, low host cell cytotoxicity, broad-spectrum activity, and minimal susceptibility to pathogen resistance, and the irrigation of wounds with PHMB has been associated with a positive healing outcome (Davis et al., 2017). The 0.1% PHMB-based wound irrigation solution demonstrated broad range antibiofilm efficacy against *P. aeruginosa*, *S. aureus,* and the multispecies biofilm, according to Salisbury et al. (2022). As for hydrogen peroxide, it is commonly used for irrigating to prevent infection, despite being associated with risks of producing oxygen emboli (Zhou et al., 2022). A synergistic approach of hydrogen peroxide and other antiseptics, such as chlorhexidine and dilute povidone-iodine, was proposed to minimize the complications associated with hydrogen peroxide but maintain the same efficacy (Flemming et al., 2016). In order to minimize the complications associated with hydrogen peroxide, our study also confirms this synergistic effect, lower concentration of hydrogen peroxide was employed in the designed irrigation solution, and a significant microbicidal effect was obtained *in vitro* and *in vivo*.

### 3.4. Histological evaluation

**Figure 4:**
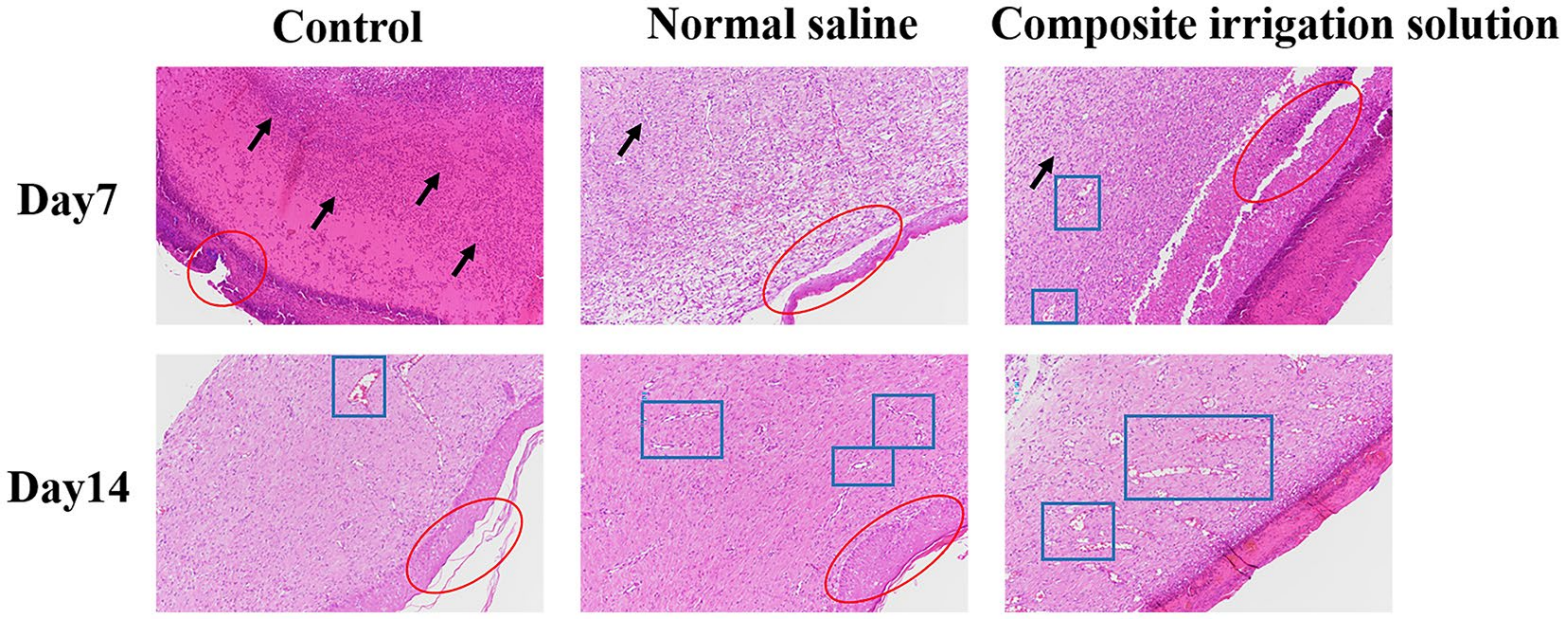
Representative H&E staining images of wound tissue in SD rats acquired on days 7 and 14 post-surgery (×100).

The histological status of different treatment groups was further assessed using hematoxylin–eosin (H&E) and Masson trichromatic staining on days 7 and 14. H&E staining can be used to evaluate the regeneration of tissue around the wound, such as inflammatory cell infiltration and fibroblast migration (Zhao and Yuan, 2022). As shown in Figure 4, the wound was still inflamed and a large number of inflammatory cells, such as neutrophils and lymphocytes (black arrow), were observed in the dermal connective tissues of the three treatment groups on the 7^th^ day. Among them, the control group exhibited more severe inflammatory cell infiltrations and fragmentary epidermal layer (red circle), and this was similar to the results of Yang et al. (2022b), in which the HE staining of the wound tissue treated by 3 M Tegaderm™ film had the longest scars and did not form re-epithelialization at day 7. In contrast, the inflammatory cell infiltration was significantly reduced in the irrigation groups, especially a small number of new blood vessels (blue boxes) had appeared in the composite irrigation solution group, indicating better wound healing. This may be attributed to the fact that the composite irrigation solution effectively removed microbes from the wound surface, avoided wound infection, and promoted blood circulation, resulting in a decrease in inflammation. This was similar to the results of Ouyang et al. (2022), where inflammatory cells were significantly reduced in the gel group due to the antibacterial effect of tetrakis(hydroxymethyl)phosphonium sulfate (THPS).

On the 14^th^ day of wound healing, the inflammatory cells were significantly reduced, while the granulation tissue was significantly increased and new blood vessels (blue box) appeared in all treatment groups, indicating that the inflammation was alleviated and the wounds were healed well (Yu et al., 2022). The composite irrigation solution group exhibited the most neovascularization, neatly aligned dermal collagen, and almost intact epithelium, while the epithelization of the control group was not completely recovered. These results indicated that the composite irrigation solution had a good anti-inflammatory effect and can promote the wound healing process. In addition to the good wound healing effect reported by Ma et al. (2022), where the CeO_2_-based dressing can almost completely repair the chronic wound infected with *Staphylococcus aureus* after 15 days, and the microalgae therapy to promote chronic wound healing by providing oxygen to the wound area (Zhong et al., 2021), inspired by the outstanding wound healing promoting effect of the irrigation solution, we believe that if the wound could be treated in time with irrigation solution before the use of these dressings, better healing can be achieved.

**Figure 5:**
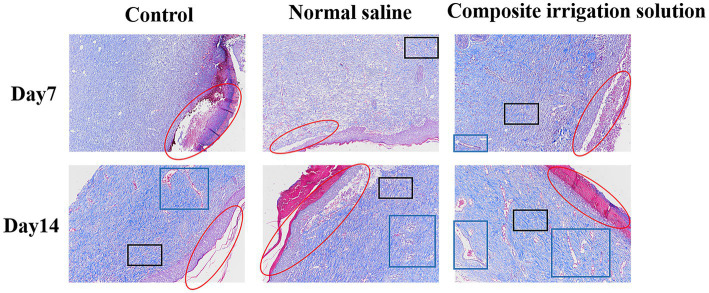
Representative Masson staining images of wound tissue in SD rats acquired on days 7 and 14 post-surgery (×100).

Masson staining is often used for differential staining of collagen fibers and muscle fibers (Deng et al., 2022), and the quantity of collagen and its maturation level can reflect the degree of wound healing. According to the Masson staining images shown in Figure 5, compared with the pronounced tissue necrosis and skin separation (red circles) in the control group at day 7, few collagen fibers (black box) were observed in the normal saline group, moderate numbers of inflammatory cells have still existed, and the collagen is lighter in color (light blue) and shows an uncompacted collagen deposition that may lead to wound contraction (He et al., 2023), while dense collagen fibers (black box) and a small number of new blood vessels (blue box) were appeared in the composite irrigation solution group, indicating that this group healed better. Normally, as the wound healing, the body secretes large amounts of collagen, which accumulates in the wound tissue and forms the restored mature skin (Yang et al., 2022a). On day 14, dense collagen fibers (black box) and blood vessels (blue box) appeared in all three groups, confirming that the wound had basically healed (Lu et al., 2017). However, although the wounds in the control and normal saline group were in the advanced stage of healing, showing obvious fibrous tissues, vascular proliferation, local tube wall thickening, few inflammatory cells, and relatively complete squamous epithelium, the separation of necrotic part and skin in the control group was still serious (red circle). In comparison, the wound healing of the composite irrigation solution group was obviously superior, where the collagen fibers were arranged neatly and distributed evenly (black box), indicating that the newly generated collagen fibers had effectively replaced the necrotic tissue (Qu et al., 2015). At the same time, the intact thick epidermis (red circle) was also observed, indicating better wound healing.

## 4. Conclusion

In this study, we explored the killing performance of the designed composite irrigation solution against the microbes common to seawater-immersed wounds and its promoting effects on wound healing. The irrigation solution exhibits an excellent bactericidal effect against *V. alginolyticus* and *V. parahaemolyticus* within 30 s, while *C. albicans*, *P. aeruginosa,* and *E. coli* can be completely killed after 1 h, 2 h, and 6 h of treatment, respectively. It also demonstrated a significant microbial reduction effect against the simulated microbial flora composed of dominant microbes in seawater-immersed wounds, and the microbes can be eradicated within 12 h of treatment. In addition, *in vivo* experiments confirmed that the composite irrigation solution had no irritation to the skin, and compared with 3 M dressing and normal saline group, the composite irrigation solution could more effectively reduce wound microbes and promote wound healing. Particularly, histological analysis showed a reduction in wound inflammatory cells while accelerating regeneration of blood vessels, collagen fibers, and epithelial tissue after treatment with the irrigation solution. In summary, these results suggested that the composite irrigation solution can be beneficial for the prevention of microbial infection related to seawater-immersed wounds, therefore, especially helpful for the wound management of the personnel who work on the sea.

## Data availability statement

The raw data supporting the conclusions of this article will be made available by the authors, without undue reservation.

## Ethics statement

The animal study was reviewed and approved by Jiangsu University (UJS-IACUC-2021041601).

## Author contributions

XW: conceptualization, methodology, writing-reviewing, and editing. JT: writing-original draft preparation. SN: experiment and data processing. DZ: experiment and data processing. BL: funding acquisition and supervision. QF: analytical guidance. All authors contributed to the article and approved the submitted version.

## Funding

This study was supported by the interdisciplinary medical and engineering research funding of the University of Shanghai for Science and Technology.

## Conflict of interest

The authors declare that the research was conducted in the absence of any commercial or financial relationships that could be construed as a potential conflict of interest.

## Publisher’s note

All claims expressed in this article are solely those of the authors and do not necessarily represent those of their affiliated organizations, or those of the publisher, the editors and the reviewers. Any product that may be evaluated in this article, or claim that may be made by its manufacturer, is not guaranteed or endorsed by the publisher.
